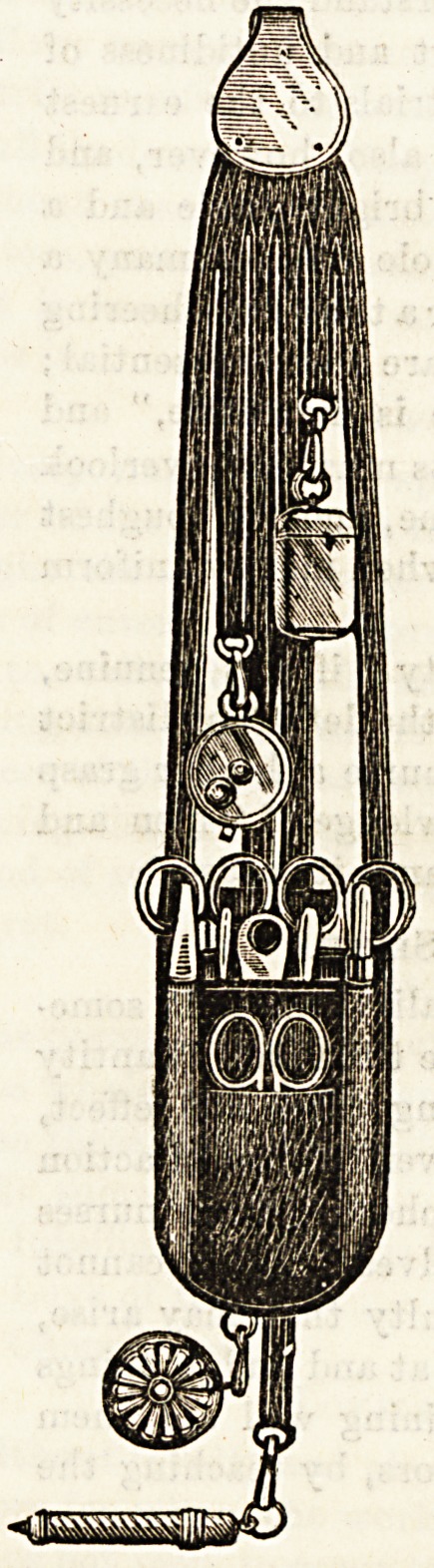# Extra Supplement.—The Nursing Mirror

**Published:** 1894-01-13

**Authors:** 


### "The Hospital" Nursing Mirror.

Being the Extra Nursing Supplement of "The Hospital" Newspaper.

[Contributions for this Supplement should be addressed to the Editor, The Hospital, 428, Strand, London, W.C., and should have the word "Nursing" plainly written in left-hand top corner of the envelope.]

### News from the Nursing World.

### A ROYAL PRESIDENT.

The new buildings in Great Portland Street, which have been erected for the National Dental Hospital and College, will be shortly opened by H.R.H. the Duke of York, who has also graciously consented to become President of the institution.

### TRAINED TO PROMPT ACTION.

Fires have recently occurred in two of the London hospitals, namely, the Victoria Chest Hospital, Victoria Park, and the Royal Free Hospital, Gray's Inn Road. Happily, no personal damage was sustained by the inmates of either of these useful institutions, and the resident medical and nursing staff appear to have acted with great promptitude and common sense. Probably it was due to everyday experience of "emergencies" that the hospital workers succeeded so well in preventing any panic amongst or injury to the patients of whom they had charge. Unluckily the nurses at the Victoria Chest Hospital have sustained serious losses by reason of the damage done to their own personal property at the time of the conflagration.

### QUEEN'S NURSES.

The Queen Victoria's Jubilee Institute has a good record of work accomplished during 1893; 125 Queen's nurses have been put on the roll; 322 are now at work in different parts of the kingdom; 39 have gained the certificates given on completion of two years' engagement to the institute. One hundred and seventy-eight local nursing associations are now affiliated to the institute; of these 75 have been affiliated during the past year. In England, 54; in Wales, 2; in Ireland, 6; and in Scotland, 13. Eighty-eight nurses have completed their training in district nursing during the year. The usual lectures on fevers, obstetrics, and hygiene have been given in the central training homes, and have been much appreciated by the nurses. The Home of Rest for Queen's Nurses at St. Katharine's has proved a most valuable possession, and has been used most advantageously during the first year of its existence. The institute now trains nurses in 9 hospitals and 18 district nursing homes. The demand for Queen's nurses is greater than the supply, as the importance of district nursing is becoming more and more felt all over the kingdom. The work demands the best nurses, those who have real love and enthusiasm for the service of the poor and suffering in this, the highest branch of the nursing profession. Those wishing for information, either as to the organising of new associations or the training of nurses, should apply to Miss Peter, Inspector of Nursing, St. Katharine's, Gloucester Gate, Regent's Park, N.W.

### POISONED BABIES.

In spite of the occasional warnings which come to the general public through the medium of inquests held on poisoned babies, it is evident that soothing syrups are still popularly regarded as "suitable nourishment" for unhappy infants. Instead of wondering at the number of deaths, we may well marvel that so many escape from the terrible risks of overdosing The regulation quantity of the narcotic is hardly likely to be accurately given by parents to whom a medicine glass is unknown. Nurses can do a great deal to discourage the use of quack medicines by losing no opportunity of pointing out the grievous injury inflicted on helpless children by these disguised poisons. District nurses have especial opportunities of giving "the word in season," which often bears good fruit long after the speaker has gone far away.

### MEDICAL WOMEN.

The decision of the British Medical Association to admit lady doctors as members has been very generally approved of. Several fully-qualified medical women are now numbered amongst the members of the Association. Before the date of the meeting at Newcastle, at which the bye-laws were altered (the words which prevented the admission of women being expunged) the matter was brought before the Sydney and New South Wales Branch, Melbourne and Victoria Branch, Adelaide and South Australian Branch, and the Cape of Good Hope Branch. It is therefore evident that in the liberal profession of medicine at any rate women have obtained all that they wish for—namely, "a fair field and no favour."

### POOR PAY.

The Sanitary Committee has recommended the Paddington Vestry to employ a lady sanitary inspector. The advantage which one would be to them in seeing that the sanitary law is carried out in those places where women only are employed is sufficiently obvious. It is a wide and valuable field of usefulness for competent women. If the Paddington Vestry consents to follow the good example already set in other places they must not deceive themselves by imagining that they can get the right class of person for the proposed £60 per annum. The position needs special education, experience, previous training in method, much tact, and intelligence in the woman who fills it. But it is hardly likely that one of the small number of ladies at present holding the diploma of the Sanitary Institute or the certificate granted at South Kensington will be willing to give her whole time to such responsible work unless it is fairly paid for. If sanitation is to take its proper place amongst the skilled work for intelligent women it must be adequately remunerated. Vestries will find reforms most imperfectly carried out by the incompetent women whom poverty not fitness will secure for their service. In inaugurating a new departure it would, however, be far better policy to offer at once such a salary as would secure the best and most suitable of qualified women inspectors.

### THE VALUE OF LABELS.

A sad event has occurred at North Kensington Infirmary through the administration of an over-dose of medicine. Ounces being the customary quantities prescribed at that institution, the head nurse, an experienced and most trustworthy woman, failed on this occasion to observe that a drachm and a half had only been ordered. The patient, who was suffering from spinal paralysis, softening of the brain, and kidney disease, has unfortunately died from the effect of the overdose of Bromidia. To invariably read the label on each bottle before giving a dose of medicine is a rule which cannot be too strongly insisted on in the training of nurses.

### PATIENTS BEFORE PROBATIONERS.

VERY sweeping censure has been meted out by Miss Gertrude Dix on the subject of the work expected of a nurse, but the rational remarks of the *British Medical Journal* are in some sort a wholesome antidote. "Facing the managers through every discussion on the subject are the facts that a considerable number of the nurses do not break down at all, even with the work they have at present, and that there are always far more candidates anxious to be trained than there are posts vacant." If writers and talkers would address their eloquence to discouraging the unsuitable women who insist upon "taking up" hospital life, they would do far better service than by wholesale condemnation of the trials, many of them imaginary, which they picture as surrounding the interesting career of a nurse. Surely all good work has its drawbacks, and every path in life its own peculiar trials, but people "without the walls" constantly fall into the mistake of thinking that hospitals exist for nurses, and not primarily for the patients, for whose benefit the public funds are contributed.

### OFFICIALLY RECOGNISED.

THE parish nurse is becoming such an acknowledged necessity that the *Lancet* even suggests the advisability of her being officially provided by means of the Local Government Board. Should such a step be taken, it will doubtless do much towards raising the status of the parish nurse. The Local Government Board is constantly found attempting to impress on guardians a fact they are slow to acknowledge—namely, that trained nurses are absolutely necessary for sick paupers. Therefore it is reasonable to hope that the standard of parish nurses will be gradually considerably raised if they obtain official recognition.

### TEA AND TALK.

EVERYONE is now familiar with the announcement that tea and coffee will be provided "after the meeting" or after the Christmas tree entertainment and many other affairs which bring people together between three and five o'clock. In fact, distinct disappointment is felt when these popular "light refreshments" are not forthcoming. Parisians appear to vary the procedure. The presence of small-pox in the gay capital has rendered vaccination fashionable, therefore invitations to tea contain the significant addition that "a cow and a doctor" will be in attendance!

### PARIS ASYLUM ATTENDANTS.

THERE has been some dissatisfaction amongst members of the nursing staff at the St. Anne Asylum in Paris because they consider that the blame attached to many of them when suicides are committed by inmates is unjustly awarded, some being punished whilst others are not. It is sad to hear that three of the unfortunate inmates have contrived to put an end to their existences in the last three months. Hitherto the average has been one a year. It is hardly surprising that under the circumstances the Director should have cautioned all the attendants to keep a strict watch on their charges. The said attendants are reported in consequence to have threatened "to strike," which is hardly a logical way of improving matters, or avoiding a recurrence of these tragedies.

### TRAINED NURSES FOR KANDY.

THE promise that trained nurses should be sent out to the hospital at Kandy, of which mention was made some time since in our columns, has been most satisfactorily carried out this week. Sister Joanna Mary, already so well known and deeply valued in Ceylon, has gone out there again, taking with her Sister Rebecca, Miss Higgins, and Miss McLaughlin, all fully-trained and experienced nurses. Our readers will join in our hearty good wishes for their safe voyage and in congratulations to the hospital which has secured such earnest and capable workers.

### A CHILDREN'S HOSPITAL.

THE Melbourne Hospital for Sick Children has recently issued its annual report, and like all other Victorian institutions it has to lament reduced means. Although salaries and expenditure have already been cut down, a further retrenchment will be required this year if the outgo is to be within the limits of the income. Unhappily the number of cases has been on the increase, the out-patients as well as the in-patients far exceeding the records of previous years. So much distress prevails amongst the poor, that the hospital at times has been called upon to give doles of food, clothing, and fuel for the use of the sick in their own homes. These gifts were supplied by the voluntary contributions of members of the committee and others. The Yorick Club, which consists of "Press-men and Literary Bohemians," gave £2 a week for some time to this fund. Certificates are granted to probationers trained at the Children's Hospital, and they have some fifty lectures delivered to them in the course of the year by the honorary and the resident medical staff. An infectious diseases hospital is urgently needed in Melbourne, and doubtless the subject will soon receive the consideration which its importance demands.

### THE ALFRED HOSPITAL, MELBOURNE.

THE Secretary and Superintendent of the Alfred Hospital has made an appeal, through the newspapers, to "Grateful patients" for help towards reducing the debt which has for a long time past seriously hampered the managers. Mr. Norman estimates that since the hospital was opened, about twenty years ago, over 20,000 in-patients have been discharged as "cured or relieved," and over 40,000 out-patients have received free advice and medicine. Of those who survive from this army of sick and disabled, few are likely to be able or willing at present to contribute; but as the typhoid season is coming on there is urgent need to reopen the wards which were closed some months ago for want of funds.

### SHORT ITEMS.

ABOUT £70 has been added to the funds of the Cold Ash Children's Cottage Hospital by means of two excellent amateur dramatic entertainments given in Newbury. The lady by whom they were arranged generously undertook to defray all incidental expenses, thus leaving the proceeds of the tickets undiminished.

### On Nursing the Nervous and Insane.

By T. DUNCAN GREENLEES, M.B.Edin., Medical Superintendent, Grahams Town Asylum, South Africa.

### I.—INTRODUCTION—THE NERVOUS SYSTEM.

To become a successful nurse of those suffering from nerve disease, and to know how to manage the mentally afflicted, requires a special education as well in these subjects as in those already acquired during the course of training previously received in a general hospital.

The asylum physician has not only to be an expert or specialist in insanity, but he is likewise required to possess a thorough knowledge of general diseases, the existence of which is not infrequently the sole cause of the mental trouble, So also the nurse who undertakes the care of nervous and mental cases should be at the same time conversant with the general principles of nursing, as well as with those rules that specially apply to this branch of her profession, and a nurse's education is incomplete unless she has undergone both a theoretical and practical training. The time has passed when a nurse was considered perfect when she knew how to make a poultice, although ignorant of the uses of moist heat, and of its physiological action on the tissues. As the field of medicine is opening up and widening for medical men, so also is the noble profession of nursing increasing its scope of usefulness, and acting as hand-maiden to progressive medicine.

My present remarks must necessarily be short and to the point. *Ars longa, vita brevis*, may well be exclaimed when studying this vast subject. I can do no more than glance superficially at the different headings, reminding readers that this is no more than an introduction to the many text books on this subject which are now published. Unfortunately these books mostly treat the matter in a disjointed and scattered way, and my endeavour will therefore be to so arrange and condense the subject as to present it in a condition for easy assimilation.

### THE NERVOUS SYSTEM.

The nervous system comprises the brain, spinal cord, nerves, and sympathetic system. The brain is a complex organ, and forms the fountain-head of all nerve action. In structure it consists of cells, fibres, and a cementing substance, the neuroglia. The cells are chiefly congregated over the surface in a layer called the cortex, and the main portion of the brain is made up of fibres and neuroglia with several masses of cells, scattered throughout, called ganglia. The cortex or grey matter presents, under the microscope, several layers of cells which are, in reality, the terminal organs of most of the nerves of the body, and any derangement in their functions likewise affects the functions of the nerves whose end organs they form. These cortical cells are connected with the fibres forming the main bulk of the brain (called the white matter), and converging towards a point at the base of the brain, cross each other before passing out, as nerves, to supply the various muscles of the body. This is called decussation of the nerve fibres, and explains the fact that any injury to the cortical cells on the one side of the brain affects the nerves and muscles on the opposite side of the body. Thus is produced "cross-paralysis." It is the fore part or frontal region of the brain that is concerned in the mental faculties; here reign supreme thought, memory, the will, and the emotions, and within those cells is evolved our intelligence. What change takes place in these little masses of protoplasm that result in the exercise of will or in the display of passions or emotions cannot be asserted to day. Science has not yet liberated the exact truth from the realms of theory on this subject.

While the frontal region forms the supreme headquarters of all thought and motion, there are other subordinate centres, as they are called, distributed over the cortex. These centres form the terminals of nerves supplying certain muscles, or groups of muscles, and special sense nerves. For example, there are centres for movements of the legs and arms, and another for the muscles of the face; likewise centres for the centres of sight, hearing, and smelling, as well as for some other functions and senses. When, however, voluntary movement is concerned then the will is cognisant, and the energy or nerve force for the movement is primarily evolved from within the frontal area of the brain cortex.

The spinal cord for the most part merely forms a medium for the conveyance of motor and sensory impulses to and from the brain. In addition to this function, however, it is capable of exercising certain functions and controlling certain movements when the brain is inert or sleeping. These functions, highly developed in the lower animals such as the frog, in man are only organic, and may be exemplified by the simple experiment of tickling the feet of a sleeping person, producing movements of the limbs.

The nerves are the telegraph wires, as it were, along which neural impulses pass from the brain to the periphery. A nerve contains two sets of fibres—motor and sensory—and therefore division of a nerve severs all connection between the brain and the parts supplied by the nerve; over this area we therefore obtain, as a result of the division of the nerve, paralysis, or loss of motion, and anæsthesia, or loss of sensation.

Should the motor fibres alone be injured, then paralysis only results, and should the sensory fibres be severed—a condition rarely found—anæsthesia alone is produced. While paralysis is frequently accompanied by loss of sensation, the latter condition is rarely met with without some motor impairment.

The sympathetic system consists of numerous groups of nerve cells and fibres arranged in masses and distributed throughout the body—the main chains being found on either side of the bodies of the vertibrae. These masses of cells, called ganglia, are likewise to be found within the walls of the heart and in the muscular coat surrounding the intestines as well as within the walls of the blood vessels. From their situations it may be inferred they are concerned in the movements of what are known as involuntary muscles—those muscles whose actions are independent of the influence of the brain. The sympathetic nerve fibres have, however, a distinct connection with the brain, and are intimately connected with it by means of the senses, as is seen in the following examples: Intense passion or emotion, arising as it does within the frontal lobe of the brain, causes flushing of the face. This flushing is due to a relaxing and consequent dilatation of the minute blood vessels which supply the skin of the face; an increased volume of blood can thereby pass through the vessels, and here we have the blushing of the countenance. Further, fear has exactly the opposite effect, constricting the blood-vessels and narrowing their lumen, a diminished supply of blood to the face produces the pallor or paleness of fear. Again, palpitation, an irregular action of the heart muscle, is frequently due simply to emotion arising within the cranial cavity, and is caused by a disturbance in the functions of the cardiac sympathetic ganglia.

We thus see how close and intimate is the connection existing between the body and the brain, between mind and matter, between physical conditions and mental states, and although a disease may not necessarily be localised within the immediate confines of the nervous system, yet so close is this connection that the brain, if not the entire nervous system, is bound to suffer from pure sympathy.

### District Nursing.

### II.—PREPARATION FOR DISTRICT NURSING.

### District versus Hospital.

In most of the training schools, both metropolitan and provincial, an impression exists that the majority of district cases being "chronic" ones, highly-trained nursing is wasted in this work. The error is due to ignorance of what is really required, and will be dispelled in time as more is known of what district nursing involves. Nurses are called upon to undertake exactly the same cases as in hospital work, with the added disadvantages of unfavourable surroundings.

### Operations.

In London and large provincial towns where hospitals and infirmaries abound, major surgical operations are not frequently attempted in the homes of the poor, though many more are accomplished than might be supposed. But in places where hospital accommodation is limited, or at too great a distance, the nurse must be prepared single-handed to attend operations, &c., for which in hospital three or four nurses are set apart. She must be well acquainted therefore with the special duties required of her, and so able to render intelligent, helpful service not only at the actual operation, but in the preparations beforehand of room and patient, and the nursing afterwards. Minor surgery in all its branches is constantly being done, and once the doctors realise the nurse is competent, much responsibility devolves upon her. She is entrusted with dressings of every kind. Her report of the case is often accepted by the busy, over-worked district doctor, and if her knowledge be limited in what she observes sad results may ensue.

### Chronic Cases.

There is no want of good work either amongst the chronic class of patients. For instance, few hospitals receive cancer in its last stages, and the relief that can be afforded to the victims of this dread disease by skilled surgical nursing can only be realised by those who have seen it. These patients need exactly the same experience in handling them and dressing their wounds as the most complicated operation case, and the more skilled the nurse the more devices she knows to give ease and comfort. District surgery calls for complete knowledge of the art of bandaging; the nurse may have to apply each and all of the many kinds, and in the case of varicose veins, ulcerated legs, &c., firm, regular pressure obtained by a properly-adjusted bandage is often half the cure. To the nurse also falls the readjustment of various splints and surgical appliances. She should be able to put on extensions, generally with only the appliances she can invent, and prepare and apply starch and gum bandages when ordered.

### Medical Nursing.

In medical work the field is wider still; thorough knowledge of the more pronounced symptoms of the diseases of the respiratory organs, cardiac, renal, and abdominal complications, throat affections, the various forms of fever, &c., is required, and to be prepared to carry out intelligently the treatment ordered by the doctor.

District nurses must understand tracheotomy cases, the giving of hot air or vapour baths to patients, in or out of bed; hot and cold packs, dry cupping, leeching, internally and externally; nasal and rectal feeding, use of catheter, and washing-out the bladder, hypodermic injections, the application and dressing of blisters, in fact, have every detail of nursing at her finger ends when called upon.

### Obstetric Work.

Knowledge of obstetric work is also essential. Nurses must be able to assist at the special operations, understand the right positions for operations and examinations, the best method of irrigation and douching, the use of the speculum and plugging with and without it, the adjustment of the various pessaries, &c.

All district nurses need not be midwives, but this particular knowledge is most valuable, especially in country posts, and at any time an emergency may arise when the knowing what to do at once may save a woman's life. Monthly nursing is indispensable; half the troubles of poor mothers arise from ignorance and neglect during the times of lying-in, and three-fourths of the blind are rendered so by want of proper attention to the new-born infant's eyes. In no other branch of nursing is the intelligent and thorough use of antiseptics more required.

### Every Day Cases.

As these different cases make up the every day work of a district nurse, the plea for complete training in every detail of her work cannot be lightly put aside. But there is danger on the other hand of every fully-trained nurse thinking she is therefore qualified for the work.

Many nurses, after varied experiences, decide to "take up district nursing" for a change, never doubting they are equal to all its demands. They obtain a post, plunge into the work without the least idea of its peculiar difficulties, and then doctors, patients and nurse suffer alike from the experiment.

It is here that the necessity of training in district work steps in.

### Necessity for Special Training.

Every nurse should spend at least six months in one or other of the established training homes where experience has taught the best lines on which to conduct the work. The idea of entering as a "probationer" repels some at first who fail to realize the different methods needed in the district. During this time, also, it can be ascertained if the physical powers of the nurse are equal to the exposure to weather and walking that is required. Every new comer goes through a period of aching feet and limbs and, generally, a severe cold at first.

### Personal Fitness.

The personal character of the nurse is really the mainspring of the whole, and comes even before her nursing abilities.

The three main essentials of a good district nurse may be briefly summed up as:—

1. Intelligent obedience to doctors' orders.
2. Love of the work.
3. Common sense.

### Loyalty to Doctors.

Without loyalty and obedience to her superior officers, the doctors for whom she works, a nurse is worse than useless. It does not seem to occur to those who disregard orders given them and apply their own remedies, that such conduct is dishonourable. The cases are under the doctor's care, and he is responsible for them; the nurse is there to carry out the treatment, and failure to do this becomes untrustworthiness.

However skilled a nurse may be, she cannot have the same practical knowledge of the conditions of the body in health and disease as the medical man, who has studied them on the dead subject as well as the living one. Yet too often the diagnosis is doubted, the treatment questioned, frequently with the audacity of inexperience. It is quite true a district nurse has often to work under practitioners who do not interest themselves greatly in their patients or use the best possible means for their relief; but that is not her business. The doctor alone is responsible, and if the nurse undertakes the case for him she must not interfere. It is a hard lesson to learn, like the question of giving relief; but disregarding it is so dangerous that too much emphasis cannot be laid upon it.

### Small Ailments.

Another temptation that arises out of highly-trained experience is that of prescribing for and treating minor ailments without a doctor. The nurse is so often asked for a remedy for some childish ailment, to treat an ulcer of long-standing, to apply poultices or other soothing applications, and it is difficult to refuse. But it should be a rule "to do nothing without doctor's orders," except under most special circumstances or in an emergency. It is very easy to fall into the habit of advising and prescribing for small ailments, but the nurse who steadily refrains is a gainer in the long run. When the doctors find she is content to remain within her own sphere, and not interfere with their work, they cease to regard her with suspicion and learn to trust her, knowing she will not try experiments upon their patients.

### Love, Tact, Courtesy, and Refinement.

Love of the work for its own sake must exist over and above the love of nursing. A district nurse needs that sympathy with her patients which enables her to fall readily into touch with them and understand their difficulties. There is so much to discourage also, unless her heart is in her work. The difficulty of making the people understand the necessity of altering many of their ways, the dirt and untidiness of the houses, the want of appliances, are trials to the earnest worker. There is plenty of brightness also, however, and she must take sunshine with her. A bright smile and a cheery word will alter the tone of a whole day for many a patient, taking them out of themselves for a time and cheering them up. Great tact and perfect courtesy are always essential; however poor, "An Englishmen's home is his castle," and nurses in their zeal to effect improvements must not overlook this. Courtesy overcomes prejudice in time, and the roughest of patients and friends insensibly soften when met by uniform pleasantness and politeness.

Refinement is another necessary quality; if not genuine, only acquired, it is apt to be laid aside in the details of district work. Educated intelligence gives the nurse a higher grasp of her profession, and the wider her knowledge of "men and manners" so much the more can she advance its cause.

### Cultivation of Common Sense.

Common sense, unfortunately, is a quality which is somewhat rare, and very few people cultivate the small quantity they possess. The difficulty in connecting cause and effect, or seeing the inevitable result of any given course of action comes to everyone in turn, and in their sphere of work nurses must be ready to think it out for themselves. There cannot be rules and regulations for every difficulty that may arise, and district nurses must learn to look at and judge things from more than one point of view. Training will aid them somewhat and prevent a repetition of errors, by teaching the result of others' experience.

### Course of Training.

Ideal preparation for district nursing would be at least two years in a large general hospital, with experience in nursing children included. Three months' district midwifery, six months' fever nursing, and a course of massage. Then at least six months in a district home where, in addition to the nursing, practical sanitation and cottage cookery were taught. Such experience, with the right character and spirit for the work, would make a district nurse whose influence would lead to the best results.

### Death in Our Ranks.

Nurse Helen Caddick died of typhoid fever, contracted in the discharge of her duties at the Queen's Hospital, Birmingham, on December 30th. She was thirty-one years of age, and had just completed her two years' probation, and was a general favourite with nurses and patients.

Nurse Ada Hunter, aged twenty, has died at the Nurses' Home, Wotton-under-Edge, of influenza. Nurse Hunter had been trained at Southend-on-Sea, and only went to Wotton a month ago.

### Royal Infirmary, Glasgow.

### THE NEW YEAR'S GATHERING OF NURSES.

"But medicine here shall ply her art
To soften pain and cheer the heart,
Shall hold with death a glorious strife,
And trim the glimmering lamp of life.

"God from His lofty throne above
Sees and approves the deed of love;
And those who misery's cry regard
Shall meet in heaven a sure reward."

### The Building.

These verses are part of a hymn composed by Mr. Hendrick, and sung ninety nine years ago at the laying of the foundation stone of the Royal Infirmary. Since that day a vast amount of good work has been accomplished within the walls. Structural improvements, and possibly new buildings, will soon become not only desirable but necessary, and doubtless the question will receive adequate consideration at the hands of the citizens of Glasgow, the Royal Infirmary having always received warm local support.

### The Nursing Staff.

The nursing school has earned a high reputation, which it will assuredly endeavour to maintain, whether the staff has to carry out its duties in old or newly-built wards. The constant applications for nurses trained at the Infirmary to fill important posts in other institutions, show that the fame of the system at Glasgow has travelled far afield. The untiring interest displayed in the education of probationers by Dr. Thomas and the Matron, Mrs. Strong, have secured an admirably-organised scheme of theoretic and practical work.

### The Annual Meeting.

The annual meeting with the nurses was held in the Dispensary of the institution, according to custom, and many friends interested in the well-being of the Royal Infirmary were present on the occasion. Amongst others, the Hon. Lord Provost Bell, who presided, Mrs. Bell, Mr. H. Brown, Sir J. King, Sir William Collins, Bailie Brechin, Deacon-Convener McLennan, Councillors Fife, Nelson, James, Dick, Mr. Nicol, City Chamberlain; Professor Macewen, Dr. Clark, Dr. Neilson, Dr. Robertson, Dr. Barlow, Dr. D. C. McVail, Mr. David M'Gowan, Mr. John E. Watson, Mr. James Thompson, Mr. Ogilvie, &c. Most affectionate reference to the late Mr. M'Ewen was made by each speaker, his death being regarded as a great public loss, and one specially felt at this annual New Year's gathering which had been inaugurated at the Royal Infirmary twenty-seven years ago by him. The Lord Provost gave an admirable address to the nurses, which was followed by Mr. Hugh Brown, chairman of the House Committee, and by Sir James King, one of the managers.

### Professor Macewen on Registration.

Professor Macewen made a particularly interesting speech, glancing first at the progress made in medicine since the first opening of the Royal Infirmary, and then passing on to the subject of nursing, in which, he said, he had always taken interest. "To a certain extent it was a selfish interest, because he knew that many operations could not be performed unless medical men had thoroughly good nurses to carry out their instructions. But a nurse was an uncertain quantity. . . . He thought there ought to be something done to remedy this state of matters. At the present time anyone could call herself a nurse, whether she had been educated or not. There was an institution, the R.B.N.A., which had for its object the registration of nurses. Personally, he had no objection to the scheme, with this exception, that if they were going to register nurses there should be some standard which would enable them to judge whether the applicant was a qualified nurse. At present they placed upon the register anybody who had been three years in a hospital. . . . Some hospitals did not pay much attention to the education of their nurses. . . . Many people said education would never make nurses; neither would mere education make doctors, but it was a help in that direction." Professor Macewen's speech was warmly applauded, and many of his hearers must have appreciated the moderation and common sense embodied in his address.

### Nursing in the United States.

### CHICAGO ALUMNÆ ASSOCIATION.

Training School Alumnæ Associations are as yet few in number, and very little is known of their work. The graduate nurses in America have hailed the opportunity to attend the course of senior lectures during this winter. This seems the best way to assist them in keeping up with the times until a post-graduate school is established. The senior lectures, given by well-known physicians, will include gynæcology, obstetrics, medical diseases, surgery, bacteriology, urinalysis, diseases of children, general lectures, nervous diseases.

### SECOND ANNUAL MEETING OF THE ILLINOIS ALUMNÆ ASSOCIATION.

Address given by Miss McIsaac, President.

The reports of the Secretary and Treasurer show a gratifying condition of our affairs. The Nursing Congress in June was a profitable lesson to all who attended. We have received four applications for information regarding our Society—from Charity, Mount Sinai, and the New York City Training Schools, all of New York; and from the Training School of the University of Pennsylvania of Philadelphia, showing that the subject of Alumnæ Associations has received an impetus.

Besides the need of organisation, we need wider intellectual range. We ought to have, we must have a code of ethics. In Article VI., Sec. I., we give to the Executive Committee power to deal with nurses whose conduct is unprofessional. Now I will ask you, how can that committee present charges and discipline without laws to govern?

Many of us are lacking in a proper professional spirit. Splendid work is often spoiled by the lack of right judgment, which might be avoided by the knowledge of a standard.

What has lifted the medical profession above the army of quacks who once pervaded this country but the combined action of their colleges and medical societies to bring about a higher standard, with a rigid code of ethics to keep men within bounds?

We want to do away with the prejudices against trained nurses. What country, what city, what profession, can be powerful or prosperous without law and order? We want to be a respected, sensible, and conservative body of women; to make our influence felt in the world. We never can do it if each member of this society runs a "go-as-you-please" race! I would like to propose a plan or outline of work for the ensuing year. I do not ask that it be accepted literally, but would suggest that some member make a motion authorising the Executive Committee to devise some such plan. I want to impress the necessity of concerted action. Our first year was a hard struggle for existence; the second has been more or less helpful. Now we are on our feet, let us make a long pull, a strong pull, and a pull all together, to give ourselves an honoured name.

My plan is as follows: First, that every graduate nurse is cordially invited to attend the lectures given to the senior class in the school. These lectures take place every Wednesday at eight p.m., from October to June, in the amphitheatre of the Cook County Hospital. A schedule of the course will be posted in the training school office for reference.

For the October meeting, that we have three papers upon the outlook for nurses in Chicago—first, by Miss Leavens, upon private duty nursing in the City, and suggestions for nurses wishing to establish themselves in smaller cities. The second, by Miss Phœbe Brown, upon home hospitals and physicians' assistants. The third, by myself, upon hospital positions, their difficulties and their future. All to be freely discussed.

For November, that Miss Hampton's paper be read and discussed. For December, that Miss Edith Campbell prepares a paper on the training school and hospital exhibits at the Fair. For January, that we have four papers upon a code of ethics; subjects, Alma mater, patients, fees, uniforms. For February, a social gathering in the evening, For March and April, discussions upon other papers from the Congress. For May, a paper upon the reading necessary for a nurse, both literary and medical. For June, banquet, with graduating class. For July, discussion of the code, and resolutions for its adoption. August, vacation. September, annual meeting, code voted upon.

Regarding our finances, we need over 4,000 dols. to endow our room. If every member would pledge to raise by her personal effort 45 dols. this year, the thing would be accomplished.

### Novelties for Nurses.

### THE "LONDON" NOISELESS CHATELAINE.

The accompanying illustration shows a most complete chatelaine for nurses, made by Mr. Stacy, of 4, Newgate Street. As will be seen at a glance, the appendages are so arranged that the unpleasant clashing so often associated with nurses' chatelaines is obviated. The instruments are all of good English make, and the "London" is supplied with all that is requisite for the surgical nurse, most conveniently arranged, and the whole appearance is elegant, neat, and workmanlike. Messrs. Stacy's catalogue shows that the most modest taste can be suited equally well with the most exacting, and we advise nurses to send for it.

### USEFUL MATERIALS.

We have received some patterns of charming materials from the Irish Industrial Fund, Donegal House, 43, Wigmore Street, W. The work done by this association is very valuable, promoting as it does native Irish industries, and securing for them a fair market. Among other warm woollen materials we specially noticed the "Hand and Heart" Homespuns, most useful for winter wear, and made to defy all weather. For underwear the "Hygeia" webbing is soft and pleasant to the touch, and cán be had in white or natural wool. Anyone requiring a very warm cloth will find the "Donegal Cloth" likely to suit their purpose, as though thick, it is wonderfully pliable in texture. For household purposes the quality of the sheetings and towellings is altogether excellent, if not exactly cheap, though some sound towelling at 6d. a yard should be specially commended. Dainty toilet slips or chairbacks might be trimmed with the pretty art laces in all varieties of harmonious shades, and the linens for art needlework are most attractive. For more strictly useful purposes there are strong and very nice crochet laces, and some of the finer hem-stitched handkerchiefs are of first-rate quality. The Donegal Industrial Fund should meet with cordial support on all hands, and we hope some of our readers may be moved to send for patterns.

### A CORRECTION.

Kent and Canterbury Institution.—The name of the Lady Superintendent is Miss Shaw, not Miss Snow, as given last week.

### Christmas Entertainments.

St. Mary's Hospital, Paddington.—The entertainment for the patients at this hospital took place on the 8th inst., and consisted of an excellent concert. A similar musical treat was provided for the nurses on the following evening.

West London Hospital.—A pleasant concert was given to the patients at this hospital on New Year's Day by the House Surgeon (Mr. Lewis) and his friends. The entertainment was much appreciated.

West Ham Hospital.—Christmas at this hospital has been observed in the usual fashion, to the delight and enjoyment of the patients. The wards were decorated by the nursing staff with appropriate mottoes, and at intervals were suspended fairy lamps. On Christmas morning the patients were each presented with a pretty card, while the little ones had playthings and picture-books. At dinner all were regaled with roast beef and Christmas pudding, and afterwards the male patients were allowed to smoke. The evening was spent in singing. The whole day was one of enjoyment and rejoicing. On the afternoon of December 27th all the patients who had been inmates for more than a month were invited to a substantial tea, provided by the Chairman of the Hospital Committee, Alderman Hay, in the hall of the dispensary. At the back of the hall there was a raised platform, and in front of it a splendid Christmas tree, decorated with useful and ornamental articles. At half-past five most of the in-patients who could be moved without danger were conveyed to the hall, and all received gifts from the Christmas tree. Many friends and supporters of the hospital came in a little later and witnessed a series of tableaux, interspersed with vocal and instrumental music, and a farce completed a very interesting entertainment.

Willesden Fever Hospital.—Special fare was provided on Christmas Day for the seventeen patients in the Willesden Fever Hospital by donations from the Harlesden tradesmen and the Constitutional Club. The kindness of friends also secured a magic lantern entertainment and distribution of toys on Boxing Day to the children, who form a large proportion of the inmates.

Queen's Hospital, Birmingham.—This hospital was prettily decorated for Christmas Day, and the dinner consisted of roast beef and plum pudding for all the patients able to partake of it. A capital entertainment took place in the evening, and the Christmas tree gifts and many valuable articles of clothing were distributed on the 3rd inst.

General Hospital, Birmingham.—Christmas Day was marked by the completion of three coloured windows for the hospital chapel, by decorations in the wards, and roast beef and plum pudding for convalescents, and chicken and custard pudding for the weakly. The usual noble Christmas tree was stripped of its attractive and useful gifts on New Year's Eve, when an entertainment took place. A Christmas tree was also provided for the children's ward.

St. Catherine's Home, Bradford.—The patients at this pretty little hospital had their party on December 28th, and each had the privilege of inviting two friends to the tempting tea which was served at five o'clock. At six a concert was given by members of the Committee and other friends, and a delightful evening was spent by the patients.

The Exeter Eye Infirmary.—Excellent Christmas fare was provided for the inmates of this hospital. Each patient had a present of clothing, the decorations were pretty, and there was some beautiful carol singing.

Devon and Exeter Hospital.—Christmas Day was a very bright festival at this hospital. The decorations were tastefully carried out, and many nice presents were provided for the patients by the kindness of the numerous friends of the popular Devon and Exeter Hospital.

West Norfolk and King's Lynn Hospital.—The Yule Tide festivities were carried out in a most pleasing manner, both for nurses and patients, under the supervision of the Matron of this hospital. The wards were suitably decorated with holly, verses, and mottoes, and on Christmas morning carols were sung in the wards. On Thursday, December 28th, 1893, about 200 out-patients sat down to tea in St. James's Hall. During this meal the police band (under the directions of Band Master Green) played selections of music, and the party finally broke up after giving three cheers for the Matron. On the following Saturday, an entertainment was given in the women's ward to the in-patients, a number of ladies and gentlemen assisting with instrumental music, songs, and recitations. After this the distribution of presents from the Christmas tree caused great amusement to the juvenile patients.

Warminster Cottage Hospital. — A very pleasant Christmas was spent at this hospital, which was prettily decorated. The children from the Orphanages of Pity sang carols in the wards. A famous Christmas tree was provided, the gifts being contributed by the local tradesmen.

Derbyshire Children's Hospital.—The Duchess of Devonshire presented a beautiful Christmas tree to the little patients, who had an enjoyable entertainment on New Year's Day. Christmas was an altogether pleasant festival in this pretty hospital.

Birmingham Royal Orthopædic and Spinal Hospital.—Former patients, as well as present ones, were entertained at this little hospital at Christmas. The lady visitors undertook the collection of presents beforehand, and the Christmas tree was well-laden. Father Christmas, duly attired, superintended the distribution of gifts after a capital tea had been enjoyed by the patients and their guests. "Box and Cox" and a selection of music were thoroughly appreciated during the evening.

Wolverhampton Workhouse Infirmary.—On Monday last, the 8th inst., the patients of the Wolverhampton Workhouse Infirmary enjoyed most heartily a "New Year's tea" of rolls and butter, various kinds of cake, jams, mince pies, oranges, &c., provided by some ladies. During the evening the patients were visited by the Messrs. Baker in nigger costume, who by their funny sayings and songs greatly added to the pleasures of the evening. The arrangements reflected great credit on the head nurse, Miss Meñou.

Edinburgh Branch, Q.V.J.I.—The Queen's Nurses in Edinburgh had 211 cases on their books on New Year's Eve, and, owing to the kind contributions of various friends, they were able to give presents to all—toys and picture books to the children, tea and sugar to the women, and tobacco to the men.

Sunderland Union Hospital.—Owing to the kindness of Miss Cowan, the Lady Superintendent, a very successful Christmas was spent at this hospital, which was very tastefully decorated by the nurses with evergreens, banners, and seasonable devices. On December 27th a tea was given to the male side, followed by a concert and dramatic entertainment by the nurses. On December 29th the same performance was given in the female wards. Many of the patients were able to be present, and thoroughly enjoyed themselves, thanks being due to the ladies and gentlemen who so kindly gave their services. On January 2nd a special tea was provided for the children, followed by a magic lantern and a Christmas tree.

The Ayr County Hospital.—Christmas keeping in Scotland is hardly on the well-established lines it is in England, and in giving the patients in a Scotch hospital a real "Merry Christmas" the matron and nursing staff have more prejudices and ignorance to combat than matrons and sisters in England would dream of. Decorations are a novelty both to patients and their friends, and carols and Christmas hymns are unfamiliar. However, in the Ayr County Hospital the day is always observed, and this Christmas each sister decorated her own wards most successfully, and the large children's ward was a perfect picture of a nursery. Each little cot was adorned with snowy muslin curtains, hung tent-wise, with a bright silver-glass ball tied with a scarlet ribbon depending from the centre of the cot, and flags and wreaths of evergreens on the walls. The long corridors, a special feature of the hospital, were regular arcades, garlands of ivy trailing across them and hanging down the walls, whilst green and crimson Chinese lanterns were suspended from the garlands. The fever blocks, though only on view through the windows, also looked gay, and the patients seemed to enjoy as merry a Christmas as those in the general building. All the patients awoke in the morning to find a parcel of useful clothing by each bed. At half-past six the sisters and nurses paraded the wards singing carols, quite a new experience for the patients, who perhaps for that reason appreciated it the more. The one o'clock dinner was given by two outside friends, and consisted of turkeys, roast beef, plum puddings, mince pies, and lemonade, followed by dessert, in each ward. After the ward dinners the servants partook of similar fare. Games and amusements went on through the afternoon, and smoking was allowed in the male wards. A few days later the large Christmas tree in the middle of the children's ward was stripped, to the delight of the children, some of whom had never seen one before. To many of the guests that night the well-kept children in their pretty white cots, in their scarlet jackets and white pinafore or toby collar, were the prettiest sight of all. There were presents for all in the hospital on the tree (for Ayrshire people are liberal to the hospital), and sweets and crackers and bright things without number. The children had toys of all sorts, a beautifully dressed doll for every girl and for the smaller boys. Convalescent patients were installed at one end of the ward, and there was a plentiful sprinkling of "daddies" from the adult wards, also of "grannies" in white night caps and scarlet nightingales. Perhaps they enjoyed it almost more than the children, for they do not have much wholesome pleasure in their hard lives. New Year's Day ended the festivities with a big tea for the patients. Some outside friends were present to see it enjoyed, an honour, however, not quite appreciated by all. One very small boy waited with apparent indifference until the last visitor had departed, then beginning his cake, remarked cheerfully and calmly, "Now we can eat—and talk."

Her Majesty's Hospital, Stepney Causeway, E.—There were a few decorations in the wards which looked very pretty and cheerful at Christmas, and the patients were much pleased at the festive preparations, to judge by the gratified excitement they exhibited over the presents placed on each bed in the early morning of the day. The usual dinner of roast beef and plum pudding was thoroughly appreciated by those able to enjoy it. Great amusement was caused by several tableaux given by the nurses, followed by Mrs. Jarley's waxworks, the intervals being filled up with suitable selections played on the pianoforte. On the 3rd inst., through the kindness of Mrs. B A. Miller, the patients who were able, numbering about 78, assembled in one ward and partook of a sumptuous tea, followed by the performances of a first-class conjurer. After this the screens were withdrawn, disclosing a prettily-decorated Christmas tree. On this was a handsome present for each patient, and attached to the present a new silver piece, 1s. to those over sixteen, and 6d. and 3d. to those under, according to age. The entertainment ended about nine p.m., after a most enjoyable time, to judge by the hearty cheers given for Mrs. Miller, who, with some of her family and friends, were present.—*Contributed.*

Glasgow Western Infirmary.—The managers of this infirmary held their annual meeting with the nurses on December 30th. Many kind speeches were made in the conservatory, to which the party adjourned. Professor Gairdner said: "It was the universal feeling of the medical staff that more than half of their work would be left unaccomplished were it not for the excellent services of the nurses." This remark was received with much applause, and the visitors and the nurses were afterwards served with afternoon tea. Earlier in the afternoon a large Christmas tree was dismantled of numerous gifts for the benefit of the little patients in the hospital. Christmas was celebrated by a series of entertainments for the benefit of the patients, nurses, and servants. On Christmas Day Miss Clyde, the Matron, was presented with a silver tea service by the nursing staff to mark the completion of her twentieth year of service. The presentation was made by Dr. Mackintosh, the medical superintendent, on behalf of the nurses, and Mr. Henry Johnston, secretary, replied for Miss Clyde. During the Christmas week the patients in most of the wards were entertained to afternoon tea and music by the lady visitors.

Sick Children's Hospital, Glasgow.—On Wednesday, by special invitation, a large company of ladies and gentlemen assembled to witness the various Christmas treats provided for the patients. The entertainments took the forms of a snowball, bran tub, magic lantern, &c., in the different wards, and all were thoroughly enjoyed by the children. It was evident to the visitors that nothing was wanting which medical skill can supply or kindness suggest to render the lot of the little sufferers as happy as possible.

Rotunda Hospital, Dublin.—This hospital was opened to the inspection of visitors interested in the work of the institution on December 23rd. After partaking of afternoon tea they went through the new and the old parts of the building. Some of the wards were prettily decorated. A number of carols were beautifully sung by some of the nurses.

National Children's Hospital.—At this admirable institution in Harcourt Street, Christmas was made a bright and happy time for the children. Toys were distributed to all, and Christmas fare to those for whom it was permitted. Many visitors assisted the kindly "Sisters" in their endeavours to make the entertainments agreeable, and their efforts were certainly crowned with success.

Meath Hospital and County Infirmary.—The patients at this hospital who were well enough to partake of it were supplied with roast beef and plum pudding on Christmas Day. Some charming toys were presented to the inmates of the children's ward by one of the Governors.

Dumfries and Galloway Royal Infirmary.—As usual, the kindly interest taken in this infirmary was shown in a practical form at Christmas. Not only was an excellent and substantial tea provided for the patients, but a good warm garment was also presented to each of them. The Lady Superintendent, Miss Hamilton, and the staff, assisted by friends, organised the pleasant entertainment, and the results were most satisfactory. The corridors were prettily decorated, and a Christmas tree was provided. Each patient was allowed to invite one friend, and the concert which followed the feast was thoroughly appreciated.

### Where to Go.

St. James's Hall.—On Friday, 12th, and Monday, 15th inst., Mrs. Longshore-Potts, M.D., will lecture to women. She is a Quakeress, and graduated from the Women's Medical College, Philadelphia. Fourteen years ago she commenced delivering lectures in drawing-rooms to women only. These were so favourably received that at the request of the Mayor of Philadelphia and others, she lectured to both sexes.

Gresham College, Basinghall Street.—Free lectures on Physic by Dr. Symes Thompson, at six p.m., on January 23rd, 24th, 25th, and 26th.

☞ **For Nurses' Looking-Glass, Book Reviews, Everybody's Opinion, and Reading to the Sick, see page cxlix et seq.**

### The Muses Looking Glass.

### AN ENGLISHMAN IN FRANCE NINETY YEARS AGO.*

At the conclusion of the Peace of Amiens, in 1802, numbers of Englishmen, anxious to see the country from which they had been severed by a ten years' war—and which, during that period, had undergone a radical change of manners and institutions—eagerly took advantage of the temporary cessation of hostilities to satisfy their curiosity by a visit to France. Among these was Mr. John Carr, a gentleman of Devonshire, who, more fortunate than many English travellers, did not remain long enough in France to be seized and imprisoned on the outbreak of a fresh war. After his return he wrote his experiences in "The Stranger in France," a quaint old book, illustrated by equally quaint engravings.

Southampton, whence he started, Mr. Carr found in a bustle, occasioned by an influx of French emigrants, who, on the conclusion of peace, began to flock into the town in great numbers to take ship for their native country. One group, our traveller's fellow passengers, was seated on the quay to look after their "aged portmanteaus" and "battered trunks," protecting themselves from the sun under umbrellas which "looked as if they had been the companions of their banishment." Among them was an old priest of more than 90 years, who, though seeming at the point of death, was undertaking the journey in the hope of ending his life in the country of his youth.

The voyage to Havre, owing to light winds, took fully two days; and when at length the vessel reached port, the passengers were rather alarmed by the huge and disorderly crowd that assembled on the quay. They were reassured, however, on discovering that it was merely the unwonted sight of a ship flying English colours, entering peacefully into their port which occasioned such a commotion among the good people of Havre.

From Havre, which seems to have been rather lacking in hotel accessories—each guest being expected to provide his own knife—our friend, after a temporary difficulty about his passport, went by Rouen to Paris. He travelled, of course, by diligence, and does not forget to note that the reports spread in England during the war of the exhaustion and commercial ruin of France were much exaggerated. He remarks, too, with satisfaction, on the passing away of the revolutionary spirit. In the year 1802 it was evidently "bad form" to be a Jacobin, and those of the "men of the Mountain" who were left had taken care to change their coats. Mr. Carr instances this by an anecdote of Santerre, the bloodthirsty brewer who presided at the execution of Louis XVI., and who about this time expressed his dislike of an acquaintance in the words, "I don't like that man; he is a Jacobin."

Naturally the First Consul, Bonaparte, the observed of all Europe, comes in for a good deal of our Englishman's attention. He saw the great man for the first time at a review, and does not seem to have been agreeably impressed, describing his countenance as "melancholy, cold, and desperate." At the opera the First Consul had a box so contrived that through the fluted pillars he could see without being seen.

Among the other notorieties, of whom our friend caught a glimpse, were Talleyrand and David—the ex-bishop at his levée, in a most unclerical costume of "embroidered scarlet, his hair full dressed, a shining sabre by his side"; and David "in his garden, putting in the background of a painting" in "a dirty robe and an old hat. His eyes" were "dark and penetrating, and beam with the lustre of genius." It was David, the regicide painter, who had persuaded the French ladies to clothe themselves in the antique dress—"if such it may be called" (as Mr. Carr says)—and indeed, from his description, undress seems the better word. We are told that David, wishing to persuade the beaux of Paris to adopt the corresponding male costume, was informed that, if he would provide a climate suitable to such light apparel, they would consider the matter. The ladies, less circumspect, braved wind and cold in the airiest of garments in order to appear statuesque; the leader of the fashion being Madame Recamier (whose identity is thinly veiled as Madame R——), who appeared one day in the Champs Elysees in a garment of so scanty a nature that even easy-going Paris was shocked, and drove her forth with hisses.

Of Joséphine Bonaparte Mr. Carr does not say much, merely remarking that, though looking older than her husband, she is "an elegant woman," and that general report speaks well of her. He saw her but once, at the opera with the First Consul, who was received "with respect, but without any clamorous acclamation."

After leaving Paris the traveller's route lay through Caen and Bayeux to Cherbourg, where he was to embark for England. His description of the combined effects of bad roads and diligence, "the ponderous machine frequently rolling from one side to the other with many alarming crackings," does not induce us to believe that we have lost much by substituting the railway for the coach. The country about Bayeux delighted our friend, reminding him of his native Devonshire. At Caen he was shown, by the custodian of Nôtre Dame, an image of the Virgin, which the same custodian had preserved for three years from Robespierre's sacrilegious agents by hiding it in his bed. It seems a pity to have to add that the image for which the poor fellow risked his life was ornamented with artificial flowers and a wig "finely curled and powdered," while the Infant Jesus in her arms was decked out in a cocked hat.

With Cherbourg Mr. Carr was disappointed. Having heard great things of the fortifications and costly works, he was surprised to find a dirty and miserable place. During his stay of two or three days he rode about the surrounding country and praises the Normandy breed of horses. A breeze having sprung up, he shipped on board a French packet and landed in Southampton, after a passage of a day and a night, feeling doubtless that he had accomplished a far greater achievement by his journey in France than his modern descendant does when he returns from a trip round the globe.

* "The Stranger in France; or Tour from Devonshire to Paris." By Mr. John Carr. Printed for J. Johnson, 72, St. Paul's Churchyard. 1803.

### Appointments.

East London Nursing Society.—Miss Cannie has been appointed Matron of the Limehouse Division of the E.L.N.S. She was trained at Marylebone Infirmary, did district nursing at Marylebone, was trained in midwifery at Chelsea, and was Superintendent of District Nursing in Londonderry. We congratulate the society on having secured so experienced a worker, and we wish Miss Cannie every success.

Hospital for Women, Brighton.—Miss M. Ord has been appointed Matron at this hospital. She was trained at the Radcliffe Infirmary, Oxford, where she afterwards held the post of Sister; and was then Charge Nurse at the Hospital for Women, at Brighton, and is to be congratulated on her promotion.

Eston Infectious Hospital.—Miss Mary Smith has received the appointment of Matron to this hospital. She was trained at the Brownlow Hill Infirmary, Liverpool, and was Charge Nurse at Netherfield Road, Liverpool, and Charge Nurse at Eastern Hospital, Homerton. We wish her every success in her new work.

### Minor Appointments.

Royal Albert Edward Infirmary, Wigan.—Miss Florence Chambers has been appointed Night Sister at this infirmary. She was trained at Guy's Hospital, where she worked for five and a-half years, and afterwards became Night Sister at St. Saviour's Infirmary, Dulwich.

### The Book World for Women and Nurses.

[We invite Correspondence, Criticism, Enquiries, and Notes on Books likely to interest Women and Nurses. Address, Editor, The Hospital (Nurses' Book World), 428, Strand, W.C.]

Dream Life and Real Life. By Ralph Iron. (London: Fisher Unwin, 1893.)

In her later works Ralph Iron contributes no rival to her early masterpiece, "The Story of an African Farm." We have looked, and looked in vain, for the fulfilment of the amazing promise her first production gave rise to, whereby she took the English reading public by storm, and where in the circumscribed space of a little volume the young writer gave voice in unparalleled directness to the great struggle which human nature combats in the tragedy of life. For to Ralph Iron when she was but a child life appeared a tragic reality, to which sentiment throughout her writings she gives direct expression, and, indeed, in this particular spirit lay the motive force of the first, as also of her later works. The hopeless despair which characterised "The African Farm" was not absent in "Dreams," an element which is all the more dominant through the strength of its literary interpretation. "Dream Life and Real Life" reflects the merits as the faults of its predecessors from the same pen. In her preface to these three short stories Ralph Iron explains she wrote the first "for a school magazine many years ago"—before, indeed, she had considered literature in any very serious light. And these tales are a triumph of pictorial simplicity, if, unfortunately, of too morbid a nature.

But in this respect the writer reflects the spirit of the times; the craze for the morbid in art and in literature abounds on all sides, a demand which has created an over-sufficient supply. And another sign of the times finds itself reflected very strongly in this writer's works, and that is the transitional phase of the epoch itself in which we live. Now an age of transition is never a becoming one; the rebellion against traditional fetters, whatever may be the ultimate good, is seldom characterized by any attractiveness at the time; and through Ralph Iron's writings this general upheaval, this restlessness, is particularly conspicuous in her female characters. Woman's mission to her is nothing if it is not revolutionary. But has not the author of "The African Farm" somewhat overstepped her limits in this respect when she puts the following words into the mouth of one of her latest heroines, who, when explaining why she despised her earliest admirers, says: "The mother heart had not swelled within me yet; I did not know all men were my children, as the large woman knows when her heart is grown." With regard to such a sentence, one can only echo what has been observed elsewhere, that one feels the only comment appropriate to such words is a fervent prayer that if we have the misfortune to know any "large" women that their hearts may not grow proportionately in that direction.

The three stories are well worth reading, as, indeed, is anything coming from Ralph Iron's pen, but one admits to a regret that the strong graphic power, the directness of expression, and literary value generally is marred, as we have already said, by the overwhelming pathos of the subject matter.

### JANUARY REVIEWS.

Many people will be amused, and perhaps more a little shocked, at Mrs. Crackanthorpe's sensational article in the Nineteenth Century, headed "The Revolt of the Daughters." It is not the overworked daughters of the East End mother who are to head this new revolt; it is the ball-going, carefully-drilled, too-little-indulged young woman in the West. We fear few people will feel called upon to pour out their sympathy on this new class of the unemployed, or care to anathematize the cruel tyranny of the mamma who thwarts them in their "innocent desire" to frequent music-halls, and make the acquaintance of casual young men disapproved of their parents. It is an acquaintance with some of the sterner facts and duties of life, not gratifying of a passing whim "to hear Chevalier sing," which will help to rescue the would-be emancipated young woman from a state of restless discontent and rebellion.

Miss Gertrude Dix delivers herself rather fiercely in the Westminster Review on the subject of "Hard Labour in the Hospitals." She argues that the whole nursing profession is labouring under the disadvantage of trying to do too much, and that the work is carried on under constant pressure, entailing an almost deadly amount of fatigue on all concerned. She would have an eight hours' day introduced into every hospital, entailing, of course, three shifts instead of two; and no doubt in places where the nature of the work imposes an exceptional strain this would be an important reform. It could not be made, however, without serious financial sacrifices. Experience certainly fails to bear out Miss Dix's contention of the unhealthiness of nursing as a profession, nor do the instances she quotes much help her case. She is particularly unfortunate in her selection of the Marylebone Infirmary for censure in nursing matters.

Miss Bulley has an interesting review in the Fortnightly on the report of the Lady Assistant Commissioners with reference to the employment of women. Miss Orme, Miss Collett, Miss Abraham, and Miss Irwine were the ladies chosen on the Commission, and as this is the first authorized investigation into the subject of woman's labour, the results are important. It is satisfactory to learn that on the whole the various trades are carried on under suitable and sanitary conditions, "a lack of thoughtfulness in minor arrangements" was noted almost everywhere, and Miss Bulley thinks that many of the small inconveniences complained of would be obviated if the wives and daughters of the manufacturers would make themselves familiar with the details of factory life, and look into such matters as the provision of proper hours and places for meals, suitable lavatories, &c., for the female hands.

### The Heroines Who Nurse Us.

*The following lines were suggested by a feeling of gratitude on the part of the parents of three children recently under treatment in the South-Western Hospital, Stockwell:—*

On every hand, in every land, in poem, song, and story,
One hears and reads the daring deeds of those who fight for glory;
On history's page, in every age, the warrior's name's recorded;
Alive or dead, to men who shed life's blood is praise awarded.
It may be well, midst scenes of hell whose light the cannon's blaze is,
With shot and shell and fiendish yell to earn both pay and praises.
One can't deny that those who die or suffer for the nation,
Who boldly fight for home and right, deserve our acclamation.
While whims of kings and other things cause battles, or incite them,
Applause is due to heroes who are brave enough to fight them.
So onward rush, with pen and brush, composer, poet, painter;
Sing on your song, both loud and long, and let it not grow fainter;
For waste of life in bloody strife, praise men—and God who gave it—
We'd rather raise a song of praise to those who strive to save it.
Turn now from sword to fever ward—clean, orderly, and quiet—
Its calm compare with din and glare in fields of wrath and riot.
Yet here, unseen, midst peace serene, a subtle foe is lurking
In vein and brain, with venomed pain, its deadly purpose working;
While a noble band, with courage grand, and disregard of dangers,
Dare hidden death in poisoned breath of fever-stricken strangers.
No crowd or crown confers renown on those who fight infection,
But we who owe to them health's glow remember with affection
The tender care of women fair, who dread Disease defeated,
Before whose arms and healing charms frustrated Death retreated.
Let those who will laud those who kill, as long as war shall curse us,
Above their cries we'll eulogise the Heroines who nurse us.

—W. P. S.

### Everybody's Opinion.

[Correspondence on all subjects is invited, but we cannot in any way be responsible for the opinions expressed by our correspondents. No communications can be entertained if the name and address of the correspondent is not given, or unless one side of the paper only be written on.]

### AN AMBULANCE SERVICE FOR PRIVATE CASES.

"A NURSE WHO WANTS INFORMATION" writes: Will you allow me to lay before your readers a householder's practical difficulty. A lady in South Kensington had a servant ill with typhlitis. General practitioner recommended immediate removal. A bed in Charing Cross Hospital was promised last Saturday, but an ambulance was necessary to get her there. The employer wired to St. John Ambulance Association. Answer: "We cannot remove typhlitis case." Knowing I was a nurse, she came to me for information. I advised the vestry; the vestry sent her to "a board of something" in West Brompton, which told her "she would not get it done in London." I then advised Hospitals Association Information Bureau, which said "Charing Cross Hospital would do it." Charing Cross Hospital said "they never did," and that St. John Ambulance did. Eventually St. John's Ambulance, on being applied to again, said "they did not remove on Saturday afternoon or Sunday (?) and thought typhlitis was an infectious disease." Having by this time (Monday afternoon) found out that it was not, the patient was removed. Now, I want to know what would have happened if this girl's mistress had not been able and willing to expend the time, money, and perseverance, which were considerable, in attaining her object. It ought not to take three days and innumerable telegrams to find out such a simple matter. I thought that the Hospitals Association had organised a system of ambulances for conveying cases of illness or accident to hospital, but I suppose I was wrong.

*** We sympathise with our correspondent, and as a practical mark of our sympathy we will supply her with the information she seeks and needs, or rather did need, for the experience she now relates should have fully informed her. But while sympathising with her in her difficulties we must take her to task for the censorious tone she adopts in speaking of Charing Cross Hospital and St. John's Ambulance Association. All her troubles were the result of her own want of knowledge, and the householder would have had no difficulty had she either sent full written particulars by messenger to the St. John Ambulance Association or, better still, have applied to them personally instead of sending a telegram. Ambulances for the transport of non-infectious cases in London are supplied only by the St. John Ambulance Association, who make a charge, the amount of which depends on the distance, and upon whether a horse ambulance or a hand ambulance is employed. The first step taken by the householder was the right one therefore, but was badly executed, as we have said. With regard to hospitals. The general hospitals of London have, most of them, their own ambulance. For very sound reasons, into which it is not necessary that we should enter, these ambulances are not available for the conveyance of patients to the hospitals, but are exclusively employed for the removal of patients from them. For infectious or contagious cases application should be made to the Metropolitan Asylums Board, whose ambulances are available for the removal of patients not only to their own hospitals, but elsewhere within the Metropolitan Asylums Board district, which extends from Highgate in the north to the Crystal Palace in the south, and from Hammersmith in the west to Abbey Wood in the east. As our correspondent mentions a vestry as an authority to which she referred the householder, we may say that, though the choice was perhaps not an unnatural one, she may spare herself the trouble in future of applying either to a vestry or board of guardians, for in the vast majority of cases they do not even provide ambulances for the proper transport of cases under their own care. "A Nurse who Wants Information" may take the fullest assurance that had the householder taken a little more pains at the commencement, she would have been spared the "time, money, and perseverance" which, as it was, she expended so freely, and three hours—not days—would have found the servant girl with typhlitis in her hospital bed.—ED. *T. H.*

### For Reading to the Sick.

### TO THE DEAR LITTLE ONES IN HOSPITAL.

WE hope you have had a happy, peaceful Cnristmas, and where it could be, a very cheerful one also. Everything has been done for you, dear children, that the love we bear you could manage, for some of you have had Christmas trees in your wards, and those who were well enough have sung—

Round about the Christmas tree,
Let us frolic blithe and free,
Singing songs of great delight
On this happy Christmas night.

You saw the pretty, bright tapers among the branches, and still prettier gifts hanging among them for every boy and girl. Tops, and horns, and dolls, and balls, and flags, with dear little woolly lambs, and oranges, and bags of sweeties. Perhaps some of you were rather frightened when the crackers went off and made a great noise, and you were glad it was only fun, and that no one was hurt. We joined in your happiness with all our hearts, but we wonder whether you know why all this rejoicing has been going on. We will tell you. It is the great feast for little children because we are keeping the birthday of our Lord Jesus Christ, who, although He was the great God who made us all, yet He came from Heaven to be a little child, just such as you yourselves are. He who was once a sweet and holy babe, now loves to see His little ones happy, and He stands among them carrying blessings in His hands,

Gifts for all who live in love,
Gifts for earth and Heaven above.

Listen to this wonderful story. A long, long time ago a beautiful infant was born of a lovely, gentle mother, who, because she had no money to pay for a room in the inn at Bethlehem, was glad to take shelter in a stable and lay her first-born in a manger. You know, I suppose, that a manger is a place which horses and cattle eat their food from, and the poor mother put her darling among the soft warm hay. And yet what a miserable bed it was for a Prince, and especially for this one, who was the son of the King of Heaven, where He had always lived in splendour with thousands of angels attending upon him; but He knew that a great many people on earth were very unhappy, so He resolved to come down as a little child and suffer Himself in the same way that they did that He might pity them still more.

First then, he was very cold and hungry, and those He came to help treated Him very unkindly, turning away His mother when she asked shelter at their doors. His mother loved Him dearly, and held Him close in her arms to comfort Him, and while He lay there a few shepherds came and worshipped Him because some angels in the heavens told them that this was the Son of God. Three very wise men also hastened from the East to bring Him presents of gold and sweet spices. But this was all the kindness He received. Very soon wicked men tried to kill Him, and His mother was obliged to carry Him away into a far country to save His life. He grew up to be the greatest Man that ever lived, helping everyone, and being kind to them, loving them all, especially little children, whom He would take into His arms and bless and embrace, bidding them to love Him as well as He loved them. So you see, however ill or weak or tired you may be, you have this kind Friend ever at hand to help you. He is the "Friend of little children," Who, though His home is "beyond the bright blue sky," yet He is about your bed, and gives His angels charge to take care of you. He puts kinds thoughts into the hearts of your friends and nurses, kind thoughts which are shown in gentle words and sweet smiles and loving care; a sevenfold blessing on themselves and on you.

Shall we not then rejoice to keep the birthday of the great King who does so much for us, and sing,

Glory to the new-born King;
Peace on earth and mercy mild,
God and sinners reconciled.

Good-bye, dear little ones, a Happy New Year to you all!

## Figures and Tables

**Figure f1:**